# Targeted detection of genetic alterations reveal the prognostic impact of H3K27M and MAPK pathway aberrations in paediatric thalamic glioma

**DOI:** 10.1186/s40478-016-0353-0

**Published:** 2016-08-31

**Authors:** Scott Ryall, Rahul Krishnatry, Anthony Arnoldo, Pawel Buczkowicz, Matthew Mistry, Robert Siddaway, Cino Ling, Sanja Pajovic, Man Yu, Joshua B. Rubin, Juliette Hukin, Paul Steinbok, Ute Bartels, Eric Bouffet, Uri Tabori, Cynthia Hawkins

**Affiliations:** 1 Arthur and Sonia Labatt Brain Tumour Research Centre, Hospital for Sick Children, Toronto, ON Canada; 2 Department of Laboratory Medicine and Pathobiology, University of Toronto, Toronto, ON Canada; 3 Division of Haematology/Oncology, Hospital for Sick Children, Toronto, ON Canada; 4 Division of Pathology, Hospital for Sick Children, Toronto, ON Canada; 5 Department of Paediatrics, Washington University School of Medicine in St. Louis, St. Louis, MO USA; 6 Division of Neurology and Oncology, Department of Paediatrics, University of British Columbia & British Columbia Children’s Hospital, Vancouver, BC Canada; 7 Division of Paediatric Neurosurgery, Department of Surgery, University of British Columbia & British Columbia Children’s Hospital, Vancouver, BC Canada; 8 Institute of Medical Science, University of Toronto, Toronto, ON Canada; 9 The Hospital for Sick Children, 555 University Avenue, Toronto, ON M5G 1X8 Canada

**Keywords:** Thalamic glioma, Pediatric, H3K27M, MAPK, BRAF, Prognostic

## Abstract

Paediatric brain tumours arising in the thalamus present significant diagnostic and therapeutic challenges to physicians due to their sensitive midline location. As such, genetic analysis for biomarkers to aid in the diagnosis, prognosis and treatment of these tumours is needed. Here, we identified 64 thalamic gliomas with clinical follow-up and characterized targeted genomic alterations using newly optimized droplet digital and NanoString-based assays. The median age at diagnosis was 9.25 years (range, 0.63–17.55) and median survival was 6.43 (range, 0.01–27.63) years. Our cohort contained 42 and 22 tumours reviewed as low and high grade gliomas, respectively. Five (12 %) low grade and 11 (50 %) high grade gliomas were positive for the H3F3A/HIST1H3B K27M (H3K27M) mutation. Kaplan-Meier survival analysis revealed significantly worse overall survival for patients harbouring the H3K27M mutation versus H3F3A/HIST1H3B wild type (H3WT) samples (log-rank *p* < 0.0001) with a median survival of 1.02 vs. 9.12 years. Mitogen-activated protein kinase (MAPK) pathway activation via BRAF or FGFR1 hotspot mutations or fusion events were detected in 44 % of patients, and was associated with long-term survival in the absence of H3K27M (log-rank *p* < 0.0001). Multivariate analysis demonstrated H3K27M status and high grade histology to be the most significant independent predictors of poor overall survival with hazard ratios of 6.945 and 7.721 (*p* < 0.0001), respectively. In contrast, MAPK pathway activation is a predictor of favourable patient outcome, although not independent of other clinical factors. Importantly, we show that low grade malignancies may harbour H3K27M mutations and that these tumours show a dismal survival compared to low grade H3WT cases. Our data strongly supports the inclusion of targeted genetic testing in childhood thalamic tumours to most accurately stratify patients into appropriate risk groups.

**Electronic supplementary material:**

The online version of this article (doi:10.1186/s40478-016-0353-0) contains supplementary material, which is available to authorized users.

## Introduction

Brain tumours are the largest group of solid tumours and the leading cause of tumour-related death in children [4]. In particular, tumours arising in midline structures of the brain including the brainstem and thalamus present significant challenges for physicians in regards to their therapeutic approach. At present, tumours arising in the brainstem, known as diffuse intrinsic pontine glioma (DIPG), have been extensively investigated and stratified into disease subtypes [6, 20]. Whereas DIPG is heavily researched, thalamic tumours, accounting for approximately 5 % of all paediatric brain neoplasms, are largely unexplored [8–10, 14, 27, 28]. Currently, the outcome of patients with thalamic tumours is predicted based on the histological grade and the potential for surgical resection of the tumour. High grade tumours classified as World Health Organization (WHO) grade III or IV are associated with worse clinical outcome as compared to grade I or II low grade tumours [23, 24]. Due to their precarious midline location and the vital functions of the thalamus and nearby structures, these tumours, particularly the diffuse gliomas, often cannot be completely resected, which is associated with a worse clinical outcome [2, 10, 22, 27]. Further, limited resections also potentially lead to sampling related errors in histological grading due to small biopsy specimens [12, 26]. As such, there is a need to identify genetic biomarkers to supplement histological grading and extent of surgical resection to aid in the diagnosis and management of thalamic tumour cases.

Recent studies have identified several biomarkers that aid in disease diagnosis and are important in predicting patient outcome in childhood gliomas. Recurrent mutations in histone H3 in which lysine 27 is substituted for methionine (H3K27M) were first described in patients with paediatric high grade glioma, primarily DIPG [20, 31, 33]. When correlated to clinical outcome, the presence of H3K27M in DIPG was associated with worse overall survival as compared to those with wild type H3 (H3WT) status, regardless of the histology of the tumour [5, 20, 21]. Subsequent studies have identified H3K27M mutations in other high grade midline tumours, including those in the thalamus, but have not looked directly at its impact on patient survival [5, 31, 33]. In addition to H3K27M, genetic aberrations affecting the RAS-MAPK pathway including KIAA1549-BRAF and other BRAF, RAF and FGFR fusion events, as well as BRAF and FGFR1 point mutations have been described primarily in hemispheric low grade gliomas in adults and children [7, 18, 19, 30, 34]. KIAA1549-BRAF fusions have previously been shown to be associated with better patient survival [3, 15] while BRAFV600E has been linked to increased likelihood of tumour progression and transformation in low grade glioma [17, 25]. FGFR1 aberrations including N546K and fusion events with TACC were reported in low grade paediatric astrocytomas [18, 34] and the former shown to be a negative prognostic marker in a small cohort of grade I pilocytic astrocytomas [3]. However, thalamic gliomas are often under-represented in glioma cohorts because their midline location often means only a biopsy is performed and there is little tissue available to study. Thus a comprehensive study of genetic markers and their role relative to histologic and clinical risk factors has not been performed for thalamic glioma. To address this limitation, we assembled a cohort of concisely defined paediatric thalamic glioma. We investigated the diagnostic and prognostic roles of defined genetic, clinical and histologic markers.

## Materials and methods

### Patient cohort

After institutional ethics board approval of the study, review of the pathology and oncology databases at the Toronto Hospital for Sick Children (SickKids) identified 101 patients diagnosed with thalamic glioma in the MRI era (1986 to 2014). As SickKids is the only reference center for children in a population of 5 million people, no selection bias is expected, and this qualifies as a population-based study. All cases were centrally reviewed for pathological diagnosis and grading according to WHO criteria (CH) [23]. Where available, MRIs were reviewed to confirm thalamic tumour origin (RK). Twenty-three tumours originally identified as thalamic were determined to not be central to or originating from the thalamus but rather, involved the thalamus and were excluded. Four cases were excluded due to bi-thalamic involvement. Ten tumours were excluded due to insufficient material yielding a final cohort of 64 (Additional file 1: Table S1). We further assembled an independent trans-Canadian cohort as previously described [32] which was used for validation purposes (Additional file 2: Table S2).

### DNA/RNA isolation

DNA was extracted from 5 to 10 10 μm thick scrolls of formalin-fixed-paraffin-embedded (FFPE) tissue using the MasterPure Complete DNA and RNA Purification Kit (Epicentre, WI, USA) according to the manufacturer’s instructions with a modified proteinase K digestion in which incubation time was increased from 24 to 48 h. Total RNA was extracted from FFPE tissue with the RNeasy FFPE extraction kit (QIAGEN, CA, USA) using the manufacturer’s guidelines. RNA/DNA quality was assessed based on 260/280 values obtained using the NanoDrop 2000 (Thermo Scientific, DE, USA) and samples between 1.7–1.9 and 1.9–2.0 were considered to be of sufficient quality for DNA and RNA, respectively. Samples were quantified using the Qubit Fluorometer V2.0 (Thermo Scientific, DE, USA).

### ddPCR mutation detection

The Bio-Rad (Hercules, CA, USA) QX200 ddPCR system was used to detect H3K27M (including H3F3A and HIST1H3B/C due to sequence similarity), H3F3A-G34V/R, BRAFV600E and FGFR1N546K mutations. Droplet digital PCR (ddPCR) workflow was completed as described [16]. Samples temporarily consisted of 1X ddPCR Supermix for Probes (no dUTP) (Bio-Rad), 900nM of specific forward and reverse primers, 250nM of specific mutant and wild type specific probe, and 10–50 ng of genomic DNA depending on the quality. Each reaction was mixed with 70 μl of Droplet Generation Oil (Bio-Rad) and partitioned into a minimum of 10,000 droplets, transferred to a 96-well plate and sealed prior to PCR amplification. PCR amplification was completed in a C1000 thermo cycler (Bio-Rad) with the following cycling conditions: 1 x (95 °C for 10 min), 39 x (95 °C for 30 s, 55 °C for 60 s, with 2 °C s^-1^ ramp rate), and 1 x (98 °C for 10 min). Following amplification, fluorescent intensity was measured with the QX200 Droplet Reader (Bio-Rad) and data analysis performed with the QuantaSoft droplet reader software (Bio-Rad). All samples were run in duplicate to ensure validity. Samples were considered positive if a minimum of 10 mutant droplet and 1 % mutant allele frequency were detected in both duplicate runs. This threshold was arbitrarily set and we report no false positive detection ever approaching this defined threshold.

The ddPCR assay was fully optimized for clinical implementation. DNA loading concentration, PCR settings, and reaction conditions were corrected to ensure sufficient probe separation for confident mutation identification (Additional file 3: Figure S1, Additional file 4: Figure S2, and Additional file 5: Figure S3). A cohort of clinical DIPG and high grade astrocytoma samples previously evaluated with whole exome sequencing (WES) (Applied Biosystems SOLiD 5500xl) were used to compare methods [20]. ddPCR showed 100 % concordance with WES calls from both fresh frozen and FFPE samples (Additional file 6: Table S3 and Additional file 7: Table S4). Importantly, it must be noted that due to sequence similarity, the H3K27M droplet digital probe is unable to distinguish between H3F3A and HIST1H3B/C, and hence will detect a K27M mutation in any of the aforementioned genes. Similar optimization procedures were completed for H3G34V/R, BRAFV600E and FGFR1N546K (data not shown). Assay sensitivity was determined by serially diluting an H3K27M-positive sample with normal DNA from 50 to 0.01 % MAF (Additional file 8: Figure S4). The ddPCR assay was able to accurately detect the mutation at allele frequencies as low as 1 % in FFPE preserved samples in accordance with the minimum detection limits described above.

### NanoString fusion detection

Probes targeting the 33 most commonly reported fusions in paediatric glioma (Additional file 9: Table S5) were designed in collaboration with NanoString (WA, USA). Five hundred nanograms of total RNA was added to the nCounter Elements TagSet in hybridization buffer and incubated at 67 °C for 20 h. The sample was processed on the nCounter Preparation Station and the cartridge scanned at 555 fields of view on the nCounter Digital Analyzer. Raw counts were subjected to a technical normalization using counts obtained for positive control probe sets included in each run. The statistical outlier detection method was used to detect the presence of an expressed fusion. Data is viewed using a box plot and the presence of an extreme outlier (3xIQR) indicate fusion expression.

### Statistics

Statistical analysis was performed using SPSS v23 (IBM Corporation) or GraphPad Prism 5 (La Jolla, CA, USA). Overall survival was determined using the Kaplan–Meier method and univariate assessments of Kaplan–Meier plots were tested using log rank. *p* values <0.05 were considered to be statistically significant. Multivariate Cox proportional hazard models and significance based on the Wald test (*α* = 0.05) were performed for multivariate analysis.

## Results

### Clinical characteristics of patients with thalamic glioma

Sixty-four patients treated at the Hospital for Sick Children from 1986 to 2014 were identified as thalamic tumour patients. Forty-two thalamic tumours were histologically diagnosed as low grade glioma (62 % grade I, 5 % grade II, and 33 % low grade glioma, NOS) whereas the remaining 22 were diagnosed as high grade glioma (41 % grade III, 50 % grade IV, and 9 % high grade, NOS). Two (5 %) low grade gliomas later transformed to high grade malignancies. Median age of diagnosis for thalamic glioma patients was 9.25 years (range, 0.63–17.55 years). Forty-one (64 %) patients received surgical resection (5 partial, 25 subtotal and 11 gross total resection) while 23 (36 %) were biopsied only. Thirty-five (55 %) and 37 (58 %) patients were treated with chemotherapy and/or radiation, respectively. Thirty-five (55 %) patients are alive (median follow-up, 12.2 years) while 29 (45 %) patients succumbed to their disease. A summary of the clinical characteristics are shown in Table 1. Clinical characteristics of the Canadian cohort reflect those described above and are available in Additional file 10: Table S6.

**Table 1:** Clinical characteristics of paediatric thalamic glioma

	Characteristic	Number of patients
Sex		
	Male	34
	Female	30
Outcome		
	Alive	35
	Dead	29
Grade		
	Low Grade	42
	High Grade	22
Histology		
	Pilocytic	23
	Diffuse	2
	Anaplastic	9
	Glioblastoma	11
	Ganglioglioma	3
	Low Grade, NOS	14
	High Grade, NOS	2
Extent of Surgery		
	GTR	11
	STR	25
	Partial Resection	5
	Biopsy	12
	Unknown	11
Radiation		
	Treated	37
	Not Treated	22
	Unknown	5
Chemotherapy		
	Treated	35
	Not Treated	26
	Unknown	3
Age at Diagnosis		
	Median	9.25 years
	Mean	8.77 ± 3.86 years
Overall Survival		
	Median	6.43 years
	Mean	8.78 ± 8.68 years

### Landscape of point mutations and fusion events in thalamic tumours

**Fig. 1:**
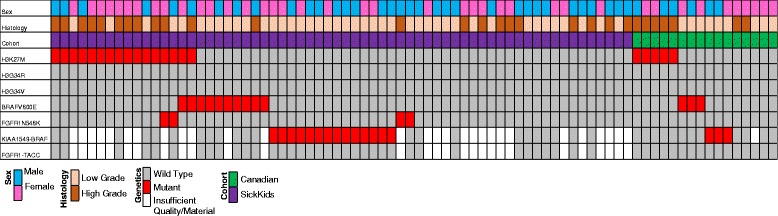
Genetic, molecular and clinical characteristics of paediatric thalamic glioma

ddPCR and NanoString assays were used to identify targeted mutations and fusions of interest based on their previous association with paediatric glioma (Fig. 1). The most recurrent hotspot mutation was H3K27M, identified in 16 (25 %) thalamic tumours tested. No H3G34R/V mutations were observed as expected. BRAFV600E mutations were present in 10 (16 %) cases, with 2 co-occurring with H3K27M mutations. KIAA1549-BRAF fusion events including those involving exons 16;09, 16;11 and 15;09 in order of prevalence, were detected in 14 (39 %) of the 36 samples from which sufficient quality RNA was obtained. These were mutually exclusive with H3K27M, BRAFV600E and FGFR fusions/mutations. FGFR1N546K mutations were seen in 4 (6 %) tumour samples. No FGFR1-TACC1, FGFR3-TACC3, other BRAF or RAF fusions or MYBL1 alterations were detected in this cohort. Within the Canadian cohort, H3K27M was identified in 5 (31 % of patients), BRAFV600E in 3 (19 %) and KIAA1549-BRAF fusion events in 3 (20 %) of 15 patients tested. No FGFR1N546K or FGFR1-TACC1, FGFR3-TACC3, other BRAF or RAF fusions or MYBL1 alterations were detected within this cohort. Genetic aberration frequencies based on histological grade can be seen in Additional file 11: Table S7.

### High grade histology and H3K27M are markers of poor prognosis in thalamic glioma

**Fig. 2:**
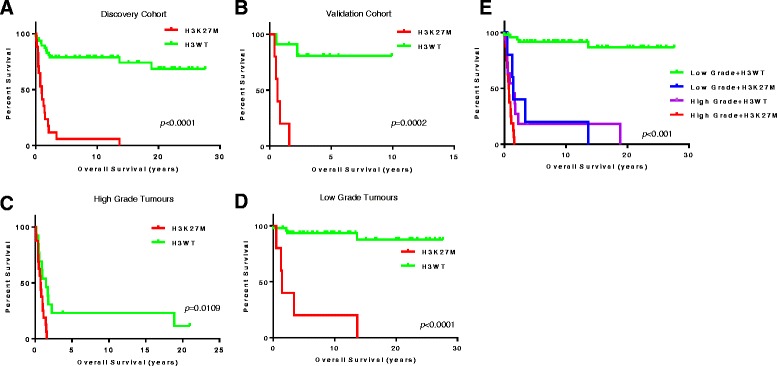
H3K27M is a negative prognostic marker in paediatric thalamic glioma. SickKids cohort (**a**) and )(**b**) Canadian cohort were tested for association between H3K27M-status and survival. Sample stratification based on histological grade showed H3K27M a significant prognostic marker in high grade (**c**) and low grade (**d**). Histology and H3K27M status combined (**e**) revealed clinical disease stratification

Tumours diagnosed as high grade glioma (grades III and IV) showed significantly worse overall survival when compared to those defined as low grade (grade I and II) in both the SickKids and Canadian cohorts (Additional file 12: Figure S5). This trend was conserved with the removal of PA and GG from the low grade histology cohort (log-rank *p* = 0.0027). 5+ year overall survival for patients diagnosed with a high grade glioma was 9.1 % (2/22) as compared to 76.2 % (32/42) in tumours with low grade histology. 9.1 % and 78.6 % of high and low grade patients respectively were alive at the time the study was completed. Likewise, the presence of H3K27M was significantly related to worse patient outcome (Fig. 2a, log-rank *p* < 0.0001). Patients harbouring the H3K27M mutation had a 5+ year overall survival of 6.3 % (1/16) as compared to 68.8 % (33/48) for H3WT patients (Table 2). Similar results were observed in the Canadian cohort (Fig. 2b, log-rank *p* = 0.0002). Of the 16 H3K27M positive cases, 5 and 11 were low and high grade glioma, respectively. All 16 of these patients succumbed to their disease. No patient with a morphologically classic pilocytic astrocytoma harboured an H3K27M mutation, suggestive of a minimum grade II histology in NOS cases.

**Table 2:** Clinical characteristics of H3K27M and H3WT paediatric thalamic glioma

	Characteristic	Number of patients	
		H3WT	H3K27M
		48	16
Sex			
	Male	28	6
	Female	20	10
Outcome			
	Alive	35	0
	Dead	13	16
Histology			
	Low Grade	37	5
	High Grade	11	11
Grade			
	Pilocytic	23	0
	Diffuse	0	2
	Anaplastic	6	3
	Glioblastoma	5	6
	Ganglioglioma	3	0
	Low Grade, NOS	11	3
	High Grade, NOS	0	2
Extent of Surgery			
	GTR	11	0
	STR	19	6
	Partial Resection	3	2
	Biopsy	7	5
	Unknown	8	3
Radiation			
	Treated	29	8
	Not Treated	16	6
	Unknown	3	2
Chemotherapy			
	Treated	29	6
	Not Treated	17	9
	Unknown	2	1
Age at Diagnosis			
	Median	9.02 years	10.50 year
	Mean	8.24 ± 3.93 years	10.35 ± 3.23 years
Overall Survival			
	Median	9.12 years	1.02 years
	Mean	11.10 ± 8.69 years	1.81 ± 3.25 years

### H3K27M is present in low grade thalamic glioma and confers a negative prognosis

When separated based on histological grade, both H3K27M and H3WT high grade glioma cases showed poor prognosis (Fig. 2c), with tumours harbouring H3K27M having slightly worse outcome (log-rank *p* = 0.0109). Long term survival (5+ years) in high grade cases was exclusively seen in H3WT patients. Strikingly, H3K27M positive low grade cases had significantly worse outcome than H3WT low grade tumours (log-rank *p* < 0.0001), with all H3K27M patients succumbing to their disease (Fig. 2d). Upon removal of PA and GG from the low grade cohort, the presence of H3K27M remained a predictor of a worse patient outcome (log-rank *p* =0.0195). Interestingly, when compared to high grade H3K27M glioma patients, patient survival was significantly longer for patients with low grade glioma histology (median survival 1.44 [range, 0.52–13.66] and 0.76 [range, 0.12–1.52] years for low grade versus high grade respectively, log-rank *p* = 0.0361) suggesting that under-grading due to sampling error does not account for the finding of H3K27M in low grade glioma. MRI review of available cases showed no obvious differences within the low grade H3K27M cases to suggest that a high grade tumour was present. Importantly, of patients with low grade thalamic gliomas and H3WT genotype, 89 % were alive at the completion of this study (median follow-up 12.2 years). Four low grade H3WT cases succumbed to their disease, all of which were WT for all aberrations tested in this study. Taken together, the presence of low grade histology and H3WT status resulted in the most favourable patient outcome, while high grade histology and H3K27M resulted in the worst outcome (Fig. 2e). Low grade histology in the presence of H3K27M as well as high grade H3WT cases act intermediately. Similar clinical outcome in H3K27M and H3WT cases were observed within the Canadian cohort (Additional file 13: Table S8).

### MAPK pathway activation is a marker of good prognosis in thalamic glioma

**Fig. 3:**
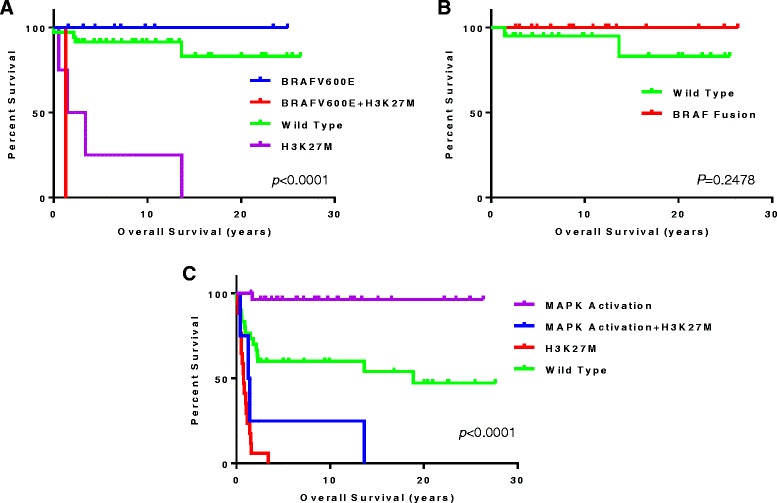
MAPK pathway activation is a positive predictive marker. BRAFV600E in the absence of H3K27M in low grade tumours. **a** shows a survival advantage. BRAF fusion events in low grade tumours. **b** shows robust survival. MAPK activation combined. **c** shows reveals a positive prognosis throughout low and high grade tumours

MAPK pathway activation via BRAF and FGFR1 hotspot mutations or fusion events was detected in 28 (44 %) of childhood thalamic gliomas. BRAFV600E was detected in 2 and 8 high and low grade gliomas, respectively. Of the 2 high grade patients with BRAFV600E, one patient also harboured H3K27M and had poor survival (1.43 years) in line with the H3K27M phenotype. Anecdotally, the patient with H3WT high grade glioma and BRAFV600E is still alive and has a longer survival compared to most other high grade tumours (3.75 years follow-up). In low grade gliomas, the presence of BRAFV600E was associated with excellent overall survival (10.27 [range, 1.27–24.95] year median survival); the only patient (1) deceased by the study endpoint was also H3K27M-positive as compared to 100 % 5-year survival of all other cases (Fig. 3a). BRAF fusion events were exclusively seen in low grade tumours, were never associated with H3K27M and were associated with excellent long-term survival (5+ year survival of 78.6 % [11/14]) (Fig. 3b). All patients harbouring a BRAF fusion event were alive upon completion of this study (mean follow-up 13.02 years). FGFR1 mutations were found in 6 % of thalamic gliomas (5 % and 9 % of low and high grade tumours respectively). FGFR1 mutations were observed to co-occur with H3K27M in two cases and as with BRAFV600E, the H3K27M phenotype of poor survival was observed. The FGFR1 positive, H3K27M negative patients behaved in accordance to their histological grade, with the grade IV case succumbing to the disease in 1.71 years while the grade I surviving 25.46 years at the time of last follow-up. FGFR1 mutations were mutually exclusive with BRAFV600E and BRAF fusion events in this series. Overall, the presence of MAPK pathway activation in the absence of H3K27M was related to robust patient survival (91 % 5-year survival), whereas in the presence of H3K27M the prognosis remained poor across all histological grades (Fig. 3c).

### H3K27M status, extent of resection and histological grade are independent predictors of patient survival in thalamic glioma

To determine their relative predictive values, we performed univariate analysis of H3K27M, MAPK pathway activation, extent of surgical resection, adjuvant therapy and histological grade. On univariate analysis histological grade (high grade versus low grade, HR 9.905 [range 4.378–22.408], *p* < 0.0001), extent of resection (resection versus biopsy, HR 0.399 [range 0.191–0.831], *p* = 0.014) and H3K27M status (K27M versus WT, HR 9.403 [range 4.253–20.788], *p* < 0.001) and MAPK pathway activation (Activated versus WT, HR 0.190 [range 0.072–0.500], *p* = 0.001 are predictive markers of overall survival (Table 3). On multivariate analysis, only H3K27M status (HR 6.945, *p* < 0.0001), histologic grade (HR 7.721, *p* < 0.0001) and extent of surgical resection (HR 0.325, *p* = 0.025) were significant independent predictors of patient survival.

**Table 3:** Univariate and multivariate Cox analysis of genetic and clinical determinants of paediatric thalamic glioma

Variable	Univariate (95 % CI)	*p* value	Multivariate (95 % CI)	*p* value
Histology (HG vs. LG)	9.905 [4.378–22.408]	<0.0001	7.721 [2.437–24.461]	<0.0001
Surgery (resection vs. biopsy)	0.399 [0.191–0.831]	0.014	0.325 [0.122–0.869]	0.025
Chemo. (yes vs. no)	0.969 [0.441–2.127]	0.969	0.549 [0.213–1.416]	0.215
Rad. (yes vs. no)	1.048 [0.459–2.396]	0.911	0.898 [0.343–2.349]	0.827
H3K27M (mut. vs. WT)	9.403 [4.253–20.788]	<0.0001	6.945 [2.190–22.020]	<0.0001
MAPK Activation (mut. vs. WT)	0.190 [0.072–0.500]	0.001	0.385 [0.134–1.102]	0.075

*LG* low grade, *HG* high grade

## Discussion

Thalamic tumours present a significant challenge for clinicians in terms of accurate diagnosis and an appropriate therapeutic approach. This study investigated the prognostic potential of molecular alterations and clinical factors in a concisely defined thalamic glioma cohort.

As expected, our results show the presence of H3K27M mutations in 50 % of high grade paediatric thalamic tumours [5, 20, 31, 33]. Interestingly, 12 % of patients diagnosed with low grade thalamic tumours also tested positive for H3K27M despite previous reports suggesting it to be exclusive to high grade cases when not located in the brainstem [31, 33]. Of note, two of five low grade cases positive for H3K27M later transformed to high grade malignancies at the time of second surgery, a rare occurrence in paediatric low grade glioma and consistent with the diagnosis of secondary HGG [25]. While it is possible that these H3K27M positive thalamic gliomas were under-graded histologically based on sampling bias, the significant survival difference observed between low grade and high grade H3K27M tumours supports the idea that these tumours were indeed distinct from their high grade counterparts. Further, there is a lack of any distinguishing MRI characteristics to suggest under-grading in these cases. Importantly, under the new World Health Organization classifications published recently, these tumours would be classified as diffuse midline glioma, H3-K27M mutant, further supporting their unique identity as compared to non-H3K27M low grade tumours [24].

Patients harbouring H3K27M showed significantly worse overall survival when compared to H3WT cases. Once separated based on histological grade, both low and high grade tumours maintained a significantly worse survival in the presence of H3K27M. Of note, high grade thalamic tumours, regardless of H3K27M status, yielded dismal survival with those harbouring the mutation succumbing to their disease in a slightly faster timeframe. This result coincides with our previous work in DIPG [5, 20], where we found H3K27M to be a negative prognostic marker in DIPG, albeit independent of tumour histology. Previous research investigating the impact of H3K27M on high grade adult midline tumours found a correlation between H3K27M and poor survival in the brainstem, but not the thalamus [1, 13]. Similar to this study, in our cohort high grade histology was associated with a poor outcome in both H3K27M and H3WT patients. However, in our cohort, several longer term survivors with H3WT high grade gliomas were present making the overall survival slightly better for H3WT patients. Work investigating paediatric glioblastoma (Grade IV) identified H3K27M positive cases as showing poor survival in midline cases including those in the brainstem and thalamic regions, consistent with our findings [21].

The presence and effect of H3K27M mutations in low grade malignancies on patient outcome has not previously been shown in malignancies outside the brainstem. In this study, patients with low grade thalamic gliomas had good overall survival with 79 % of patients alive upon the completion of this study (mean follow-up 14.03 years), consistent with previous studies [3, 11, 15, 29, 30]. However, all 5 patients whose low grade gliomas were positive for H3K27M succumbed to their disease, with increased latency compared to high grade H3K27M cases. These findings substantiate the knowledge that H3K27M mutations do exist in low grade tumours and that H3K27M status can supplement histological grading in determining the clinical progression of the tumour. Importantly, the poor survival of patients with H3K27M low grade glioma suggests that they should be treated as high grade glioma; receiving more aggressive adjuvant therapies than would be typically given to low grade glioma patients.

MAPK pathway activation was detected in 44 % of patients. BRAFV600E was detected in 10 (16 %) of the thalamic tumour samples, a slightly lower percentage than previously reported [29, 30]. However, in these reports, the thalamus was grouped with all diencephalon-based structures, potentially altering the true prevalence. Furthermore, this study identified the presence of BRAFV600E mutations in 3 grade I malignancies for the first time, in contrast with previous reports [11]. In the absence of H3K27M, BRAFV600E cases were associated with a good clinical outcome. Previously we reported that low grade tumours having BRAFV600E were at a higher risk for developing secondary high grade glioma [25]. However, only 3 patients within this initial study were identified as thalamic, of which one co-occurred with H3K27M and another with CDKN2A deletion, potentially predisposing to tumour transformation. Furthermore, although the results here suggest robust survival in our BRAFV600E cohort, our median follow-up was 10.27 years and it is possible that with longer latency these patients may develop high grade glioma. Therefore, patients harboring low grade BRAFV600E tumours should be monitored closely post-treatment for disease progression. In the presence of H3K27M, BRAFV600E patients had an outcome similar to BRAFWT, H3K27M-positive cases, suggesting that H3K27M is the dominant prognostic indicator. KIAA1549-BRAF fusion events were exclusive to low grade thalamic glioma and were found in 39 % of tested tumours. This percentage is similar to previous reports in midline paediatric low grade glioma [11, 15]. However, it should be noted that these studies included non-thalamic locations, including the cerebellum, hypothalamus, optic pathway and brainstem. Patients with a KIAA1549-BRAF fusion showed robust survival, with all 14 patients alive at the time this study was completed (median follow-up: 11.55 years). FGFR1N546K was detected in 4 patients (6 %) and was seen in both low and high grade cases. FGFR1N546K mutations have been previously linked to decreased patient survival [3]. The small number of patients harbouring this mutation in this cohort means conclusions related to its prognostic significance cannot be drawn. However, of the 4 FGFR1 mutated patients, the 2 also harbouring H3K27M showed poor survival in accordance with the H3K27M phenotype. Alternately, FGFR1 mutated patients without H3K27M behaved in accordance with their histological grade, suggesting that histology and H3K27M status are the main phenotypic contributors in these cases. We did not identify FGFR1-TACC1 or FGFR3-TACC3 fusions in our cohort as described in [34].

The presence of MAPK pathway activation was found in 28 patients within our cohort, 89 % of which were low grade glioma. Importantly, of the 3 high grade cases with MAPK activation, 2 were also H3K27M and behaved in accordance with the H3K27M phenotype. The remaining high grade, MAPK activated sample shows a prolonged survival in comparison to other high grade tumours (3.75 years) and was alive upon the completion of this study. In low grade tumours, the presence of MAPK activation, regardless of fusion or hotspot mutation event was related to prolonged survival. Taken together, H3K27M and MAPK pathway activation effectively stratifies thalamic tumours into survival groups. MAPK pathway activation in the absence of H3K27M confers long-term survival across the entire cohort irrespective of tumour histology. Tumours wild type for the genetic targets tested here behave in close accordance with their histological grade. Further testing is required to identify additional genetic marks capable of further stratifying this group. Importantly, it must be recognized that a limitation of this study in respect to MAPK activation is the inclusion of pilocytic astrocytoma and ganglioglioma within the low grade histology category. This saturation of circumscribed and non-invasive lesions positive for MAPK activation may partially explain the robust survival seen. However, in the case of thalamic tumours, the finding appear consistent across histological grades and as such, remain an important clinical predictor of patient outcome. Lastly, tumours harbouring H3K27M, regardless of histology or MAPK activation show dismal survival. In this respect, H3K27M is one of the most critical factors in predicting patient outcome in thalamic glioma cases and must be considered equally important as tumour histology in primary prognostic categorization.

## Conclusions

In summary, this study supports the inclusion of targeted H3K27M and MAPK activation testing via clinically approved methodology in all paediatric midline tumours regardless of histological grade. This helps provide an accurate prediction of patient outcome and appropriate therapeutic options. Further, we provide fully optimized and cost-effective strategies to targeting these aberrations in a clinical setting.

## Additional files

Additional file 1: Table S1. SickKids cohort details. (XLSX 17 kb) Additional file 2: Table S2. Canadian cohort details. (XLSX 11 kb) Additional file 3: Figure S1. Droplet digital PCR minimum concentration detection. (PPTX 55 kb) Additional file 4: Figure S2. Droplet digital PCR annealing temperature comparison. (PPTX 173 kb) Additional file 5: Figure S3. Droplet digital PCR pre-PCR digestion versus no digestion comparison. (PPTX 102 kb) Additional file 6: Table S3. Droplet digital PCR H3K27M detection validation as compared to WES and Sanger Sequencing. (DOCX 12 kb) Additional file 7: Table S4. Droplet digital PCR H3K27M detection validation raw droplet counts. (XLSX 12 kb) Additional file 8: Figure S4. Droplet digital PCR minimum mutant allele frequency detection. (PPTX 38 kb) Additional file 9: Table S5. NanoString low grade glioma fusion and duplication detection panel. (XLSX 10 kb) Additional file 10: Table S6. Clinical characteristics of paediatric thalamic glioma Canadian cohort. (DOCX 12 kb) Additional file 11: Table S7. Histology Kaplan-Meier survival analysis within the A) SickKids cohort and B) Canadian cohort. (DOCX 12 kb) Additional file 12: Figure S5. Clinical characteristics of H3K27M and H3WT paediatric thalamic glioma Canadian cohort. (PPTX 84 kb) Additional file 13: Table S8. Clinical characteristics of H3K27M and H3WT paediatric thalamic glioma in the Canadian cohort. (DOCX 12 kb)

## References

[1] Aihara K, Mukasa A, Gotoh K, Saito K, Nagae G, Tsuji S, Tatsuno K, Yamamoto S, Takayanagi S, Narita Y, Shibui S, Aburatani H, Saito N (2014). H3F3A K27M mutations in thalamic gliomas from young adult patients. Neuro Oncol.

[2] Baroncini M, Vinchon M, Mineo JF, Pichon F, Francke JP, Dhellemmes P (2007). Surgical resection of thalamic tumours in children: approaches and clinical results. Child’s Nerv Syst.

[3] Becker AP, Scapulatempo-Neto C, Carloni AC, Paulino A, Sheren J, Aisner DL, Musselwhite E, Clara C, Machado HR, Oliveira RS, Neder L, Varella-Garcia N, Reis RM (2015). KIAA1549:BRAF gene fusion and FGFR1 hotspot mutations are prognostic factors in pilocytic astrocytomas. J Neuropathol Exp Neurol.

[4] Bouffet E, Capra M, Bartels U (2009). Salvage chemotherapy for metastatic and recurrent ependymomas of childhood. Child’s Nerv Syst.

[5] Buczkowicz P, Bartels U, Bouffet E, Becher O, Hawkins C (2014). Histopathological spectrum of paediatric diffuse intrinsic pontine glioma: diagnostic and therapeutic implications. Acta Neuropathol.

[6] Buczkowicz P, Hoeman C, Rakopoulos P, Pajovic S, Letourneau L, Dzamba M, Morrison A, Lewis P, Bouffet E, Bartels U, Zuccaro J, Agnihotri S, Ryall S, Barszczyk M, Chornenkyy Y, Bourgey M, Bourque G, Montpetit A, Cordero F, Castelo-Branco P, Mangerel J, Tabori U, Ho KC, Huang A, Taylor KR, Mackay A, Bendel AE, Nazarian J, Fangusaro JR, Karajannis MA, Zagzag D, Foreman NK, Donson A, Hegert JV, Smith A, Chan J, Lafay-Cousin L, Dunn S, Hukin J, Dunham C, Scheinemann K, Michaud J, Zelcer S, Ramsay D, Cain J, Brennan C, Souweidane MM, Jones C, Allis CD, Brudno M, Becher O, Hawkins C (2014). Genomic analysis of diffuse intrinsic pontine gliomas identifies three molecular subgroups and recurrent activating ACVR1 mutations. Nat Genet.

[7] Chi AS, Batchelor TT, Yang D, Dias-Santagata D, Borger DR, Ellisen LW, Iafrate AJ, Louis DN (2013). BRAF V600E mutation identifies a subset of low-grade diffusely infiltrating gliomas in adults. J Clin Oncol.

[8] Colosimo C, di Lella GM, Tartaglione T, Riccardi R (2002). Neuroimaging of thalamic tumours in children. Child’s Nerv Syst.

[9] Cuccia V, Monges J (1997). Thalamic tumours in children. Child’s Nerv Syst.

[10] Di Rocco C, Iannelli A (2002). Bilateral thalamic tumours in children. Child’s Nerv Syst.

[11] Faulkner C, Ellis HP, Shaw A, Penman C, Palmer A, Wragg C, Greenslade M, Haynes HR, Williams H, Lowis S, White P, Williams M, Capper D, Kurian KM (2015). KIAA:1549-BRAF 15-9 fusions are more frequent in the midline than within the cerebellum. J Neuropathol Exp Neurol.

[12] Feiden W, Steude U, Bise K, Gundisch O (1991). Accuracy of stereotactic brain tumour biopsy: comparison of the histologic findings in biopsy cylinders and resected tumour tissue. Neurosurg Rev.

[13] Feng J, Hao S, Pan C, Wang Y, Wu Z, Zhang J, Yan H, Zhang L, Wan H (2015). The H3.3 K27M mutation results in a poorer prognosis in brainstem gliomas than thalamic gliomas in adults. Hum Pathol.

[14] Fernandez C, Maues de Paula A, Colin C, Quilichini B, Bouvier-Labit C, Girard N, Scavarda D, Lean G, Figarella-Branger D (2006). Thalamic gliomas in children: an extensive clinical, neuroradiological and pathological study of 14 cases. Child’s Nerv Syst.

[15] Hawkins C, Walker E, Mohamad N, Zhang C, Jacob K, Shirinian M, Alon N, Kahn D, Fried I, Scheinemann K, Tsangaris E, Dirks P, Tressler R, Bouffet E, Jabado N, Tabori U (2011). KIAA1549-BRAF fusion predicts better clinical outcome in paediatric low-grade astrocytoma. Clin Cancer Res.

[16] Hindson BJ, Ness KD, Masquelier DA, Belgrader P, Heredia NJ, Makarewicz AJ, Bright IJ, Lucero MY, Hiddessen AL, Legler TC, Kitano TK, Hodel MR, Petersen JF, Wyatt PW, Steenblock ER, Shah PH, Bousse LJ, Troup CB, Mellen JC, Wittman DK, Erndt NG, Cauley TH, Koehler RT, So AP, Dube S, Rose KA, Montesclaros L, Wang S, Stumbo BP, Hodges SP, Romine S, Milanovich FP, White HE, Regan JF, Karlin-Neumann GA, Hindson GM, Saxonov S, Colston BW (2011). High-throughput droplet digital PCR system for absolute quantitation of DNA copy number. Anal Chem.

[17] Horbinski C, Nikiforova MN, Hagenkord JM, Hamilton RM, Pollack IF (2012). Interplay among BRAF, p16, p53, and MIB1 in paediatric low-grade gliomas. Neuro Oncol.

[18] Jones DT, Hutter B, Jager N, Korshunov A, Kool M, Warnatz HJ, Zichner T, Lambert SR, Ryzhova M, Quang DA, Fontebasso AM, Stutz AM, Hutter S, Zuckermann M, Sturm D, Gronych J, Lasitschka B, Schmidt S, Seker-Cin H, Witt H, Sultan M, Ralser M, Northcott PA, Hovestadt V, Bender S, Pfaff E, Stark S, Faury D, Schwartzentruber J, Majewski J, Weber UD, Zapatka M, Raeder B, Schlesner M, Worth CL, Bartholomae CC, von Kalle C, Imbusch CD, Radomski S, Lawerenz C, van Sluis P, Koster J, Volckmann R, Versteeg R, Lehrach H, Monoranu C, Winkler B, Unterberg A, Herold-Mende C, Milde T, Kulozik AE, Ebinger M, Schuhmann MU, Cho YJ, Pomeroy SL, von Deimling A, Witt O, Taylor MD, Wolf S, Karajannis MA, Eberhart CG, Scheurlen W, Hasselblatt M, Ligon KL, Kieran MW, Korbel JO, Yaspo ML, Brors B, Felsberg J, Reifenberger G, Collins VP, Jabado N, Eils R, Lichter P, Pfister SM, International Cancer Genome Consortium PedBrain Tumour Project (2013). Recurrent somatic alterations of FGFR1 and NTRK2 in pilocytic astrocytoma. Nat Genet.

[19] Jones DT, Kocialkowski S, Liu L, Pearson DM, Backlund LM, Ichimura K, Collins VP (2008). Tandem duplication producing a novel oncogenic BRAF fusion gene defines the majority of pilocytic astrocytomas. Cancer Res.

[20] Khuong-Quang DA, Buczkowicz P, Rakopoulos P, Liu XY, Fontebasso AM, Bouffet E, Bartels U, Albrecht S, Schwartzentruber J, Letourneau L, Bourgey M, Bourque G, Montpetit A, Bourret G, Lepage P, Fleming A, Lichter P, Kool M, von Deimling A, Sturm D, Korshunov A, Faury D, Jones DT, Majewski J, Pfister SM, Jabado N, Hawkins C (2012). K27M mutation histone H3.3 defines clinically and biologically distinct subgroups of paediatric diffuse intrinsic pontine glioma. Acta Neuropathol.

[21] Korshunov A, Ryzhova N, Hovestadt V, Bender S, Sturm D, Capper D, Meyer J, Schrimpf D, Kool M, Northcott PA, Zheludkova O, Milde T, Witt O, Kulozik AE, Reifenberger G, Jabado N, Perry A, Lichter P, von Deimling A, Pfister SM, Jones DT (2015). Integrated analysis of paediatric glioblastoma reveals a subset of biologically favourable tumours with associated molecular prognostic markers. Acta Neuropathol.

[22] Kramm KC, Butenhoff S, Rausche U, Warmuth-Metz M, Kortmann RD, Pietsch T, Gnekow A, Jorch N, Janssen G, Berthold F, Wolff JE (2011). Thalamic high-grade gliomas in children: a distinct clinical subset?. Neuro Oncol.

[23] Louis DN, Ohgaki H, Wiestler OD, Cavenee WK, Burger PC, Jouvet A, Scheithauer BW, Kleihues P (2007). The 2007 WHO classification of tumours of the central nervous system. Acta Neuropathol.

[24] Louis DN, Perry A, Reifenberger G, von Deimling A, Figarella-Branger D, Cavenee WK, Ohgaki H, Wiestler OD, Kleihues P, Ellison DW (2016). The 2016 World Health Organization Classification of Tumours of the Central Nervous System: a summary. Acta Neuropathol.

[25] Mistry M, Zhukova N, Merico D, Rakopoulos P, Krishnatry R, Shago M, Stavropoulos J, Alon N, Pole JD, Ray PN, Navickiene V, Mangerel J, Remke M, Buczkowicz P, Ramaswamy V, Guerreiro Stucklin A, Li M, Young EJ, Zhang C, Castelo-Branco P, Bakry D, Laughlin S, Shlien A, Chan J, Ligon KL, Rutka JT, Dirks PB, Taylor MD, Greenberg M, Malkin D, Huang A, Bouffet E, Hawkins CE, Tabori U (2015). BRAF mutation and CDKN2A deletion define a clinically distinct subgroup of childhood secondary high-grade glioma. J Clin Oncol.

[26] Mittler M, Walters MD, Stopa EG (1996). Observer reliability in histological grading of astrocytoma stereotactic biopsies. J of Neurosurg.

[27] Ozek MM, Ture U (2002). Surgical approach to thalamic tumours. Child’s Nerv Syst.

[28] Puget S, Crimmins DW, Garnett MR, Grill J, Oliveira R, Boddaert N, Wray A, Lelouch-Tubiana A, Roujeau T, Di Rocco F, Zerah M, Sainte-Rose C (2007). Thalamic tumours in children: a reappraisal. J Neurosurg.

[29] Schiffman JD, Hodgson JG, VanderBerg SR, Flaherty P, Polley MY, Yu M, Fisher PG, Rowitch DH, Ford JM, Berger MS, Ji H, Gutmann DH, James CD (2010). Oncongenic BRAF mutation with CDKN2A inactivation is characteristic of a subset of paediatric malignant astrocytomas. Cancer Res.

[30] Schindler G, Capper D, Meyer J, Janzarik W, Omran H, Herold-Mende C, Schmieder K, Wesseling P, Mawrin C, Hasselblatt M, Louis DN, Korshunov A, Pfister S, Hartmann C, Paulus W, Reifenberger G, von Deimling A (2011). Analysis of BRAF V600E mutation in 1,320 nervous system tumours reveals high mutation frequencies in pleomorphic xanthoastrocytoma, ganglioglioma and extra-cerebellar pilocytic astrocytoma. Acta Neuropathol.

[31] Schwartzentruber J, Korshunov A, Liu XY, Jones DT, Pfaff E, Jacob K, Sturm D, Fontebasso AM, Quang DA, Tönjes M, Hovestadt V, Albrecht S, Kool M, Nantel A, Konermann C, Lindroth A, Jäger N, Rausch T, Ryzhova M, Korbel JO, Hielscher T, Hauser P, Garami M, Klekner A, Bognar L, Ebinger M, Schuhmann MU, Scheurlen W, Pekrun A, Frühwald MC, Roggendorf W, Kramm C, Dürken M, Atkinson J, Lepage P, Montpetit A, Zakrzewska M, Zakrzewski K, Liberski PP, Dong Z, Siegel P, Kulozik AE, Zapatka M, Guha A, Malkin D, Felsberg J, Reifenberger G, von Deimling A, Ichimura K, Collins VP, Witt H, Milde T, Witt O, Zhang C, Castelo-Branco P, Lichter P, Faury D, Tabori U, Plass C, Majewski J, Pfister SM, Jabado N (2012). Driver mutations in histone H3.3 and chromatin remodelling genes in paediatric glioblastoma. Nature.

[32] Steinbok P, Gopalakrishnan CV, Hengel AR, Vitali AM, Poskitt K, Hawkins C, Drake J, Lamberti-Pasculli M, Ajani O, Hader W, Mehta V, McNeely PD, McDonald PJ, Ranger A, Vassilyadi M, Atkinson J, Ryall S, Eisenstat DD, Hukin J (2016). Paediatric thalamic tumours in the MRI era: a Canadian persecptive. Childs Nerv Syst.

[33] Wu G, Broniscer A, McEachron TA, Lu C, Paugh BS, Becksfort J, Qu C, Ding L, Huether R, Parker M, Zhang J, Gajjar A, Dyer MA, Mullighan CG, Gilbertson RJ, Mardis ER, Wilson RK, Downing JR, Ellison DW, Zhang J, Baker SJ, St. Jude Children’s Research Hospital–Washington University Paediatric Cancer Genome Project (2012). Somatic histone H3 alterations in paediatric diffuse intrinsic pontine gliomas and non-brainstem glioblastomas. Nat Genet.

[34] Zhang J, Wu G, Miller CP, Tatevossian RG, Dalton JD, Tang B, Orisme W, Punchihewa C, Parker M, Qaddoumi I, Boop FA, Lu C, Kandoth C, Ding L, Lee R, Huether R, Chen X, Hedlund E, Nagahawatte P, Rusch M, Boggs K, Cheng J, Becksfort J, Ma J, Song G, Li Y, Wei L, Wang J, Shurtleff S, Easton J, Zhao D, Fulton RS, Fulton LL, Dooling DJ, Vadodaria B, Mulder HL, Tang C, Ochoa K, Mullighan CG, Gajjar A, Kriwacki R, Sheer D, Gilbertson RJ, Mardis ER, Wilson RK, Downing JR, Baker SJ, Ellison DW, St. Jude Children’s Research Hospital–Washington University Paediatric Cancer Genome Project (2010). Whole-genome sequencing identifies genetic alterations in paediatric low-grade glioma. Nat Genet.

