# Supplementary material for: Targeted detection of genetic alterations reveal the prognostic impact of H3K27M and MAPK pathway aberrations in paediatric thalamic glioma
Source: Acta Neuropathol Commun. 2016 Aug 31;4(1):93. doi: 10.1186/s40478-016-0353-0 (PMC5006436; doi:10.1186/s40478-016-0353-0)
Supplement: Additional file 3: Figure S1. — Droplet digital PCR minimum concentration detection. (PPTX 55 kb) [file 40478_2016_353_MOESM3_ESM.pptx]

## Slide 1
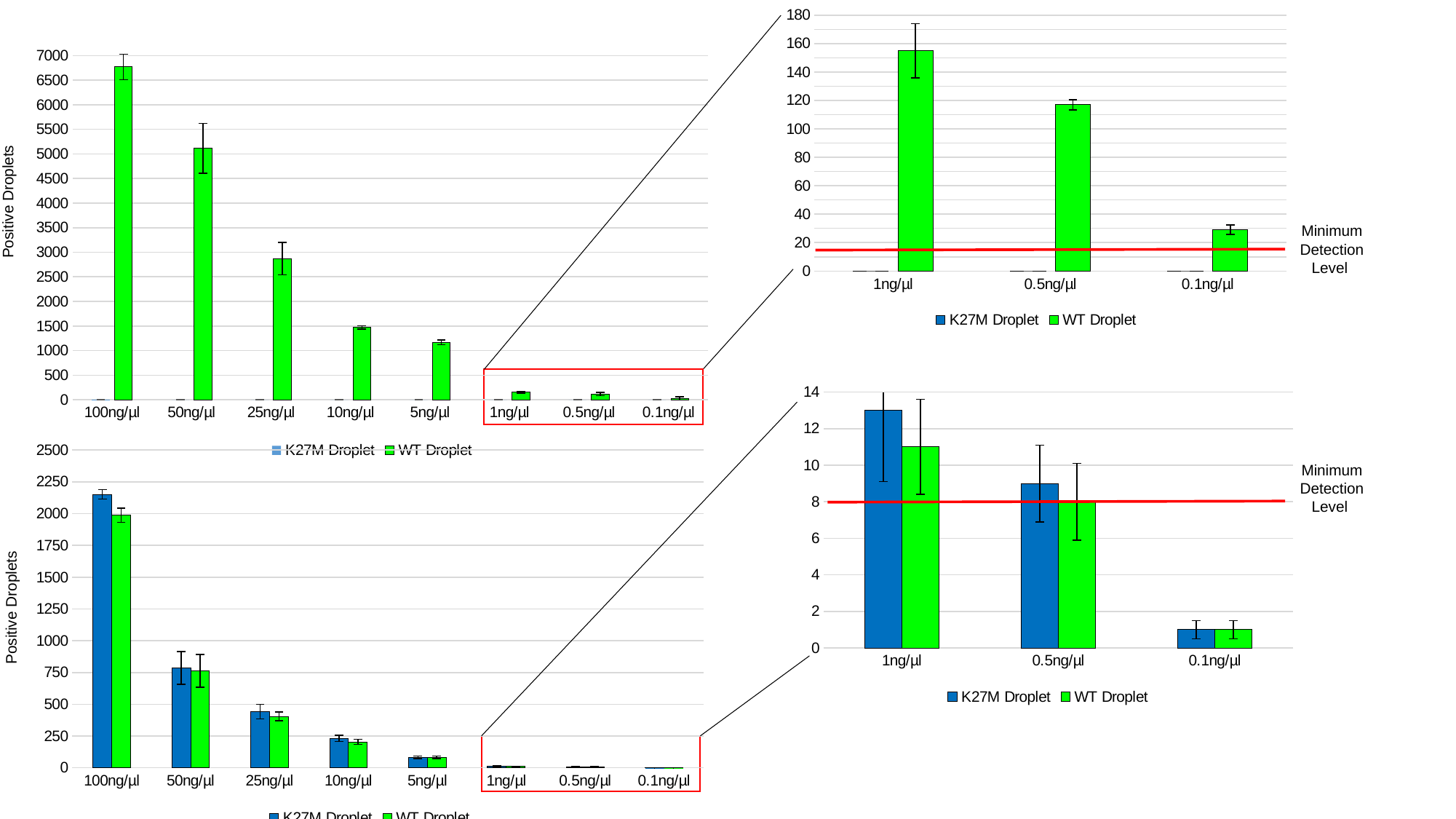

### Chart
| Category | K27M Droplet | WT Droplet |
|---|---|---|
| 1ng/µl | 0.0 | 155.0 |
| 0.5ng/µl | 0.0 | 117.0 |
| 0.1ng/µl | 0.0 | 29.0 |
### Chart
| Category | K27M Droplet | WT Droplet |
|---|---|---|
| 100ng/µl | 3.0 | 6769.0 |
| 50ng/µl | 0.0 | 5113.0 |
| 25ng/µl | 0.0 | 2869.0 |
| 10ng/µl | 0.0 | 1472.0 |
| 5ng/µl | 0.0 | 1164.0 |
| 1ng/µl | 0.0 | 155.0 |
| 0.5ng/µl | 0.0 | 117.0 |
| 0.1ng/µl | 0.0 | 29.0 |Positive Droplets
Minimum Detection Level
### Chart
| Category | K27M Droplet | WT Droplet |
|---|---|---|
| 1ng/µl | 13.0 | 11.0 |
| 0.5ng/µl | 9.0 | 8.0 |
| 0.1ng/µl | 1.0 | 1.0 |
### Chart
| Category | K27M Droplet | WT Droplet |
|---|---|---|
| 100ng/µl | 2151.0 | 1987.0 |
| 50ng/µl | 785.0 | 762.0 |
| 25ng/µl | 442.0 | 404.0 |
| 10ng/µl | 231.0 | 204.0 |
| 5ng/µl | 83.0 | 82.0 |
| 1ng/µl | 13.0 | 11.0 |
| 0.5ng/µl | 9.0 | 8.0 |
| 0.1ng/µl | 1.0 | 1.0 |Minimum Detection Level
Positive Droplets
