# Supplementary figures and images for: Targeted detection of genetic alterations reveal the prognostic impact of H3K27M and MAPK pathway aberrations in paediatric thalamic glioma
Source: Acta Neuropathol Commun. 2016 Aug 31;4(1):93. doi: 10.1186/s40478-016-0353-0 (PMC5006436; doi:10.1186/s40478-016-0353-0)

## Slide 1
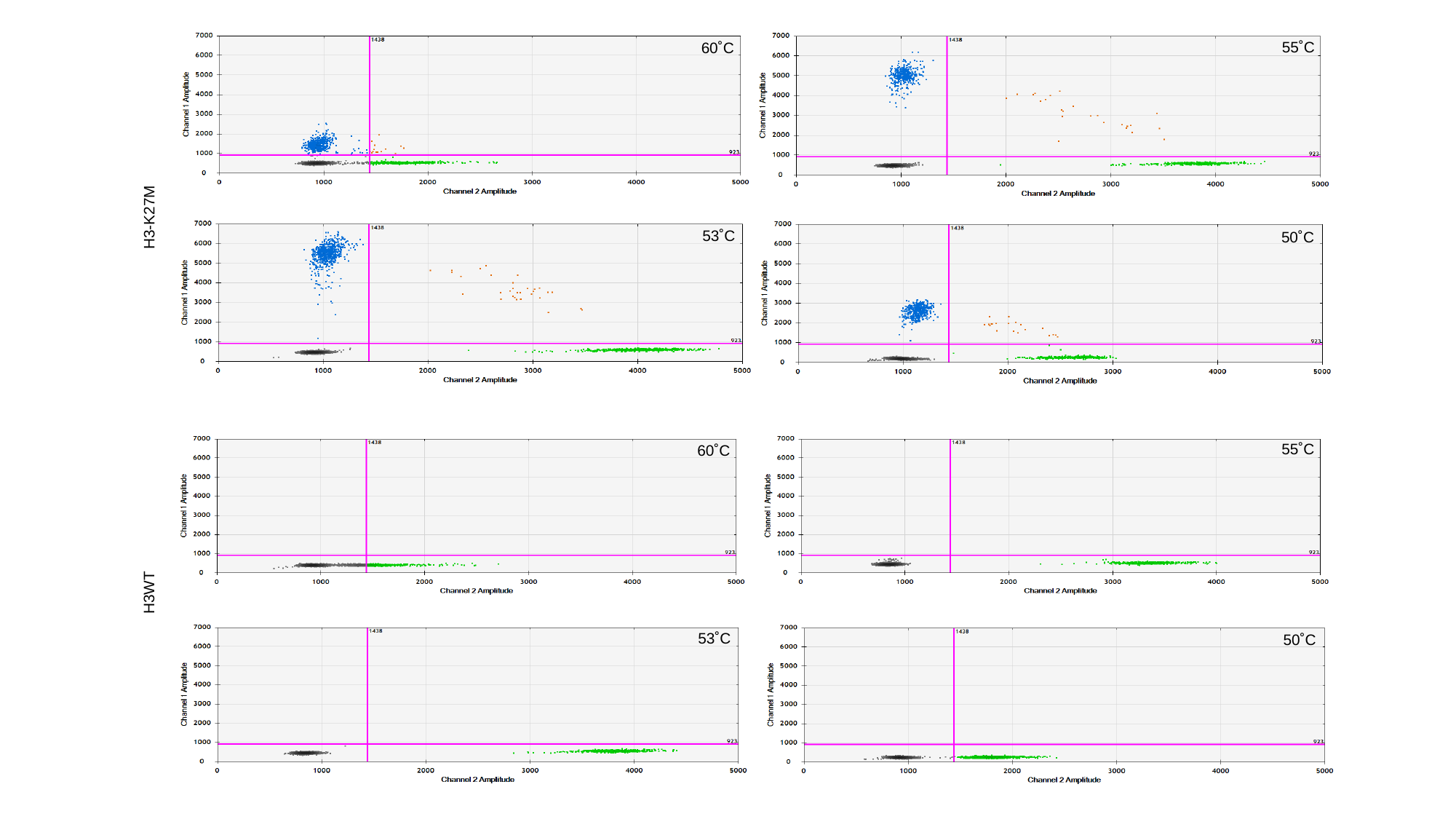

55˚C
60˚C
53˚C
50˚C
H3-K27M
55˚C
60˚C
53˚C
50˚C
H3WT

Supplement: Additional file 4: Figure S2. — Droplet digital PCR annealing temperature comparison. (PPTX 173 kb) [file 40478_2016_353_MOESM4_ESM.pptx]

## Slide 1
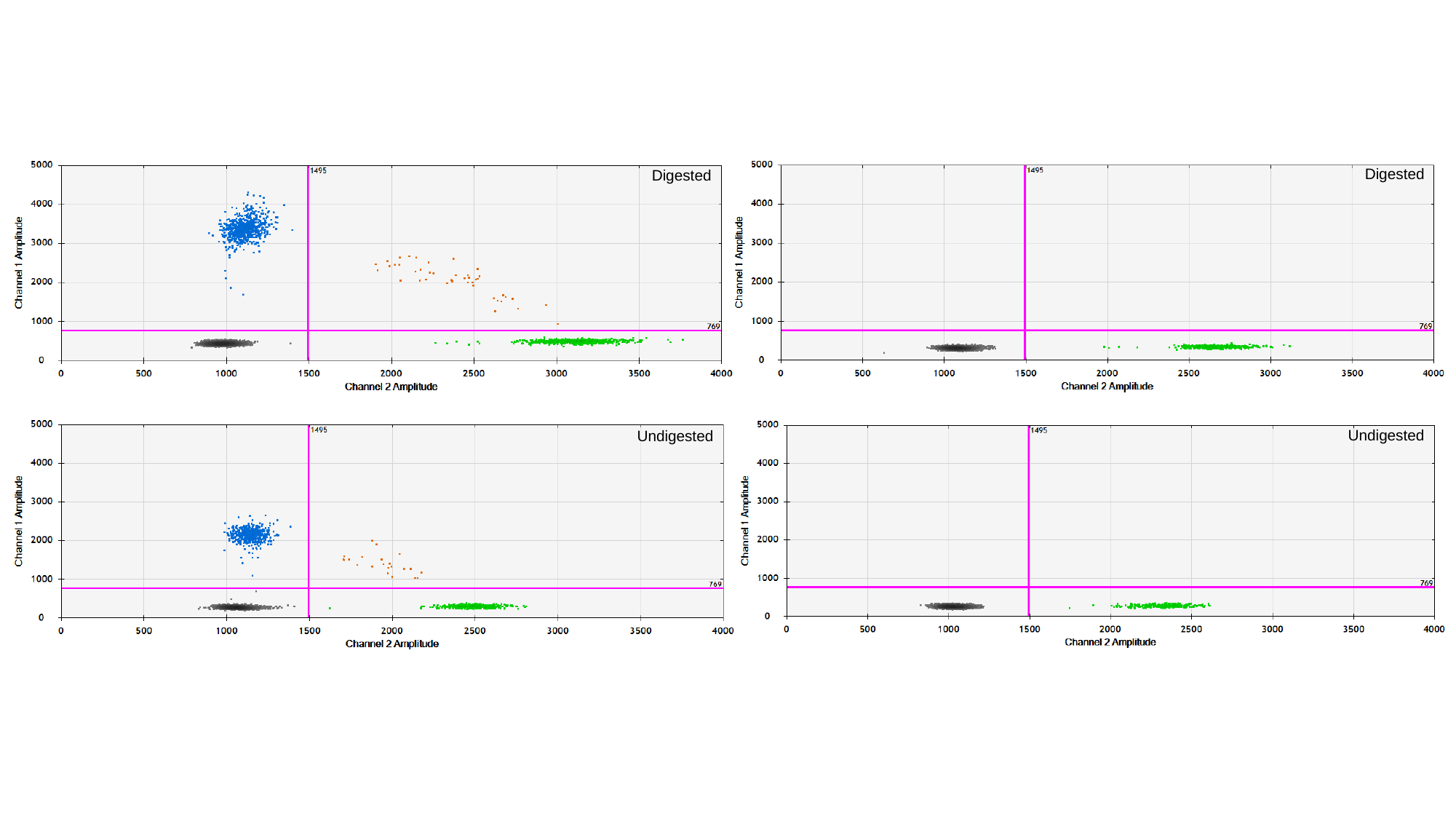

Digested
Digested
Undigested
Undigested

Supplement: Additional file 5: Figure S3. — Droplet digital PCR pre-PCR digestion versus no digestion comparison. (PPTX 102 kb) [file 40478_2016_353_MOESM5_ESM.pptx]

## Slide 1
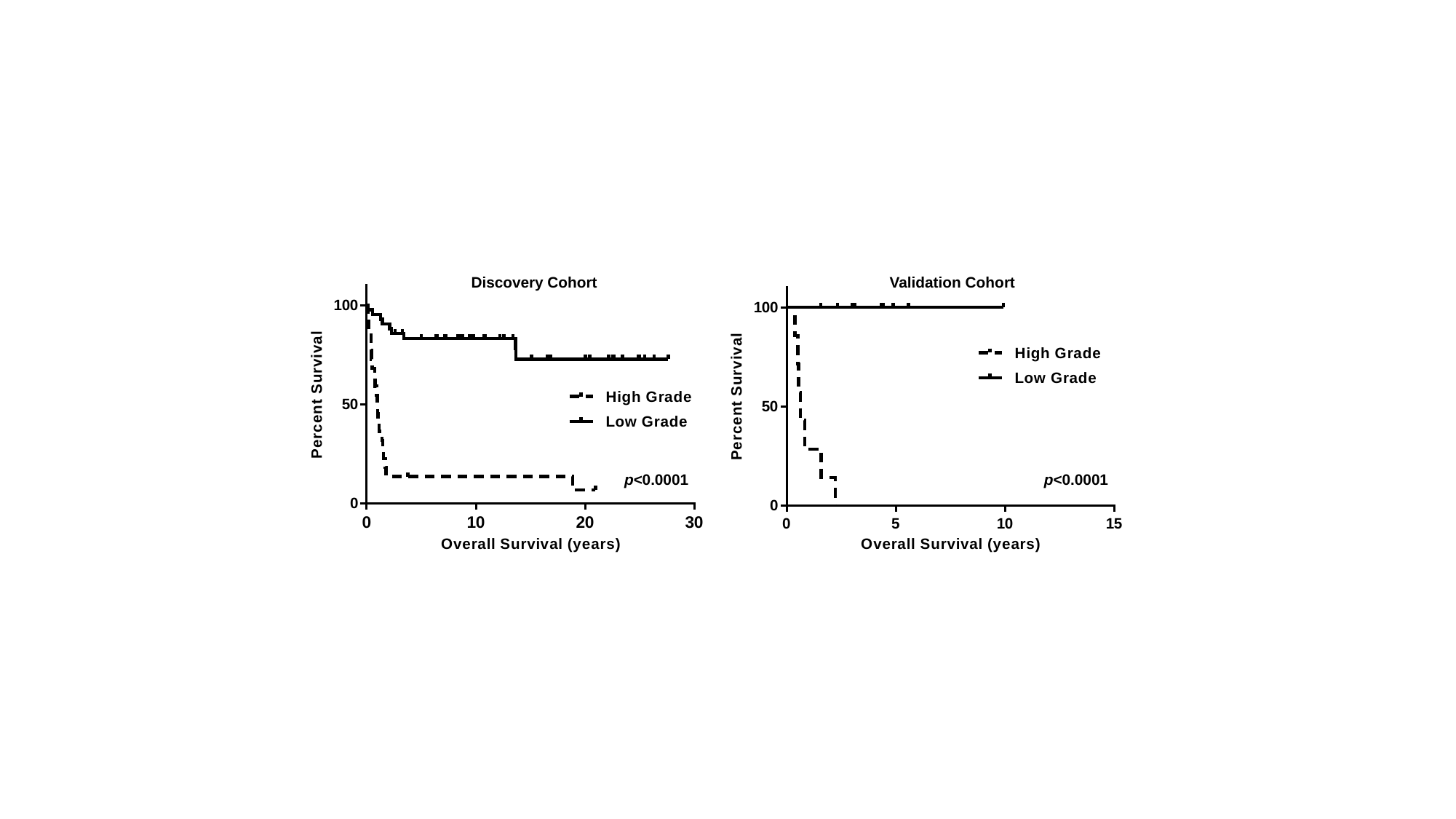

Discovery Cohort
Validation Cohort
p<0.0001
p<0.0001

Supplement: Additional file 12: Figure S5. — Clinical characteristics of H3K27M and H3WT paediatric thalamic glioma Canadian cohort. (PPTX 84 kb) [file 40478_2016_353_MOESM12_ESM.pptx]
