# Supplementary material for: Targeted detection of genetic alterations reveal the prognostic impact of H3K27M and MAPK pathway aberrations in paediatric thalamic glioma
Source: Acta Neuropathol Commun. 2016 Aug 31;4(1):93. doi: 10.1186/s40478-016-0353-0 (PMC5006436; doi:10.1186/s40478-016-0353-0)
Supplement: Additional file 6: Table S3. — Droplet digital PCR H3K27M detection validation as compared to WES and Sanger Sequencing. (DOCX 12 kb) [file 40478_2016_353_MOESM6_ESM.docx]

| **Sample ID** | **Mutation Detection Method** | | |
| --- | --- | --- | --- |
|  | **Sanger** | **WES/WGS** | **ddPCR** |
| DIPG03 | H3.3WT | H3.3WT | H3WT |
| DIPG06 | H3.3K27M | H3.3K27M | H3K27M |
| DIPG07 | H3.3K27M | H3.3K27M | H3K27M |
| DIPG09 | H3.3WT | H3.3WT | H3WT |
| DIPG14 | Test Failed | H3.3K27M | H3K27M |
| DIPG19 | **H3.3WT** | **H3.3K27M** | **H3K27M** |
| DIPG25 | H3.3K27M | H3.3K27M | H3K27M |
| DIPG26 | H3.3K27M | H3.3K27M | H3K27M |
| DIPG28 | H3.3K27M | H3.3K27M | H3K27M |
| DIPG29 | H3.3WT | H3.3WT | H3WT |
| DIPG30 | H3.3K27M | H3.3K27M | H3K27M |
| DIPG62 | Test Failed | H.3.1K27M | H3K27M |
| HGA36 | H3.3K27M | H3.3K27M | H3K27M |
| HGA38 | H3.3K27M | H3.3K27M | H3K27M |
| HGA50 | H3.3K27M | H3.3K27M | H3K27M |
| HGA55 | H3.3WT | H3.3WT | H3WT |
| HGA56 | H3.3WT | H3.3WT | H3WT |
| HGA69 | H3.3K27M | H3.3K27M | H3K27M |
| HGA75 | H3.3K27M | H3.3K27M | H3K27M |
