# Supplementary material for: Targeted detection of genetic alterations reveal the prognostic impact of H3K27M and MAPK pathway aberrations in paediatric thalamic glioma
Source: Acta Neuropathol Commun. 2016 Aug 31;4(1):93. doi: 10.1186/s40478-016-0353-0 (PMC5006436; doi:10.1186/s40478-016-0353-0)
Supplement: Additional file 8: Figure S4. — Droplet digital PCR minimum mutant allele frequency detection. (PPTX 38 kb) [file 40478_2016_353_MOESM8_ESM.pptx]

## Slide 1
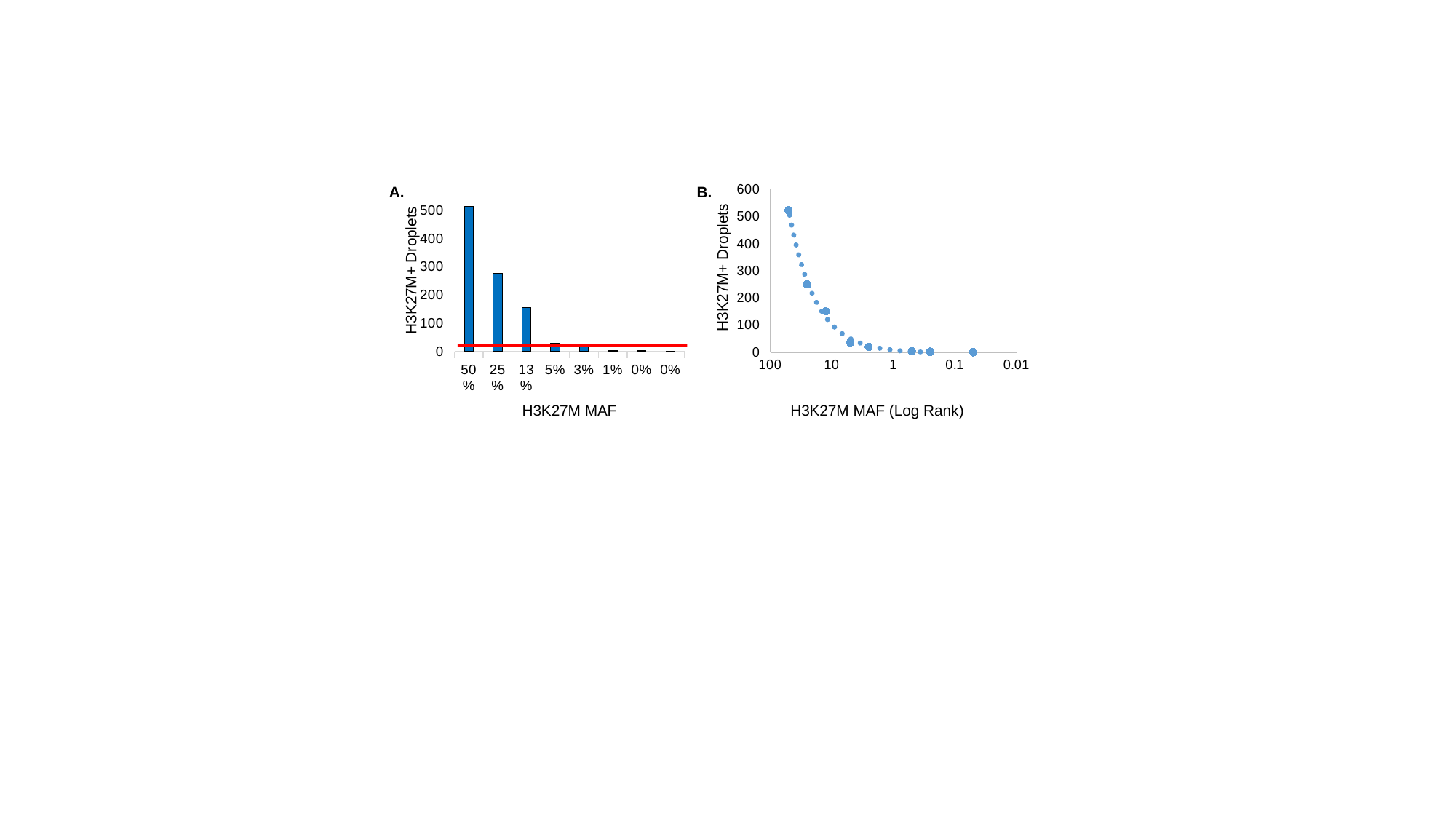

### Chart
| Category | Positive Droplets |
|---|---|
### Chart
| Category | Positive Droplets |
|---|---|
| 0.5 | 516.0 |
| 0.25 | 278.0 |
| 0.125 | 155.0 |
| 0.05 | 28.0 |
| 2.5000000000000001E-2 | 19.0 |
| 5.0000000000000001E-3 | 4.0 |
| 2.5000000000000001E-3 | 2.0 |
| 5.0000000000000001E-4 | 1.0 |B.
A.
H3K27M+ Droplets
H3K27M+ Droplets
H3K27M MAF (Log Rank)
H3K27M MAF
