# Supplementary material for: Targeted detection of genetic alterations reveal the prognostic impact of H3K27M and MAPK pathway aberrations in paediatric thalamic glioma
Source: Acta Neuropathol Commun. 2016 Aug 31;4(1):93. doi: 10.1186/s40478-016-0353-0 (PMC5006436; doi:10.1186/s40478-016-0353-0)
Supplement: Additional file 10: Table S6. — Clinical characteristics of paediatric thalamic glioma Canadian cohort. (DOCX 12 kb) [file 40478_2016_353_MOESM10_ESM.docx]

|  | Characteristic | Number of Patients |
| --- | --- | --- |
| Sex |  | |
|  | Male | 6 |
|  | Female | 10 |
| Outcome |  | |
|  | Alive | 9 |
|  | Dead | 7 |
| Histology |  | |
|  | Low Grade | 9 |
|  | High Grade | 7 |
| Grade |  | |
|  | Pilocytic | 6 |
|  | Diffuse | 3 |
|  | Anaplastic | 5 |
|  | Glioblastoma | 2 |
|  | Ganglioglioma | 0 |
|  | Low Grade, NOS | 0 |
|  | High Grade, NOS | 0 |
| Extent of Surgery |  | |
|  | GTR | 4 |
|  | STR | 9 |
|  | Partial Resection | 1 |
|  | Biopsy | 0 |
|  | Unknown | 2 |
| Radiation |  | |
|  | Treated | 9 |
|  | Not Treated | 7 |
|  | Unknown | 0 |
| Chemotherapy |  | |
|  | Treated | 9 |
|  | Not Treated | 7 |
|  | Unknown | 0 |
| Age at Diagnosis |  | |
|  | Median | 8.57yrs |
|  | Mean | 8.80 ± 4.80yrs |
| Overall Survival |  | |
|  | Median | 2.29yrs |
|  | Mean | 2.87 ± 2.53yrs |
