# Supplementary material for: Targeted detection of genetic alterations reveal the prognostic impact of H3K27M and MAPK pathway aberrations in paediatric thalamic glioma
Source: Acta Neuropathol Commun. 2016 Aug 31;4(1):93. doi: 10.1186/s40478-016-0353-0 (PMC5006436; doi:10.1186/s40478-016-0353-0)
Supplement: Additional file 11: Table S7. — Histology Kaplan-Meier survival analysis within the A) SickKids cohort and B) Canadian cohort. (DOCX 12 kb) [file 40478_2016_353_MOESM11_ESM.docx]

|  | **PA** | **GG** | **DA** | **LGG. NOS** | **AA** | **GBM** | **HGG, NOS** |
| --- | --- | --- | --- | --- | --- | --- | --- |
| **H3K27M** | 0 | 0 | 2 | 3 | 6 | 8 | 2 |
| **H3G34R/V** | 0 | 0 | 0 | 0 | 0 | 0 | 0 |
| **BRAFV600E** | 3 | 2 | 3 | 3 | 1 | 1 | 0 |
| **FGFR1N546K** | 1 | 0 | 1 | 0 | 0 | 2 | 0 |
| **BRAF Fusion** | 15 | 0 | 0 | 2 | 0 | 0 | 0 |
