# Supplementary material for: Targeted detection of genetic alterations reveal the prognostic impact of H3K27M and MAPK pathway aberrations in paediatric thalamic glioma
Source: Acta Neuropathol Commun. 2016 Aug 31;4(1):93. doi: 10.1186/s40478-016-0353-0 (PMC5006436; doi:10.1186/s40478-016-0353-0)
Supplement: Additional file 13: Table S8. — Clinical characteristics of H3K27M and H3WT paediatric thalamic glioma in the Canadian cohort. (DOCX 12 kb) [file 40478_2016_353_MOESM13_ESM.docx]

|  | Characteristic | Number of Patients | |
| --- | --- | --- | --- |
|  |  | H3WT | H3K27M |
|  |  | 11 | 5 |
| Sex |  | | |
|  | Male | 3 | 3 |
|  | Female | 8 | 2 |
| Outcome |  | | |
|  | Alive | 9 | 0 |
|  | Dead | 2 | 5 |
| Histology |  | | |
|  | Low Grade | 9 | 0 |
|  | High Grade | 2 | 5 |
| Grade |  | | |
|  | Pilocytic | 6 | 0 |
|  | Diffuse | 3 | 0 |
|  | Anaplastic | 2 | 3 |
|  | Glioblastoma | 0 | 2 |
|  | Ganglioglioma | 0 | 0 |
|  | Low Grade, NOS | 0 | 0 |
|  | High Grade, NOS | 0 | 0 |
| Extent of Surgery |  | | |
|  | GTR | 4 | 0 |
|  | STR | 6 | 3 |
|  | Partial Resection | 1 | 0 |
|  | Biopsy | 0 | 0 |
|  | Unknown | 0 | 2 |
| Radiation |  | | |
|  | Treated | 7 | 2 |
|  | Not Treated | 4 | 3 |
|  | Unknown | 0 | 0 |
| Chemotherapy |  | | |
|  | Treated | 7 | 2 |
|  | Not Treated | 4 | 3 |
|  | Unknown | 0 | 0 |
| Age at Diagnosis |  | | |
|  | Median | 6.19yrs | 11.66yrs |
|  | Mean | 8.09 ± 5.35yrs | 10.37 ± 3.26yrs |
| Overall Survival |  | | |
|  | Median | 3.14yrs | 0.63yrs |
|  | Mean | 3.82 ± 2.53yrs | 0.79 ± 0.48yrs |
